# The Impact of Lipoprotein Apheresis on Oxidative Stress Biomarkers and High-Density Lipoprotein Subfractions

**DOI:** 10.1155/2020/9709542

**Published:** 2020-08-05

**Authors:** Agnieszka Mickiewicz, Ewelina Kreft, Agnieszka Kuchta, Ewa Wieczorek, Joanna Marlęga, Agnieszka Ćwiklińska, Milena Paprzycka, Marcin Gruchała, Marcin Fijałkowski, Maciej Jankowski

**Affiliations:** ^1^1st Department of Cardiology, Medical University of Gdansk, Gdansk, Poland; ^2^Department of Clinical Chemistry, Medical University of Gdansk, Gdansk, Poland

## Abstract

Lipoprotein apheresis (LA) treatment results in a substantial reduction of low-density lipoprotein- (LDL-) cholesterol and lipoprotein(a) concentrations, which consequently decreases the rate of cardiovascular events. The additional benefit of LA may be associated with its impact on the composition and quality of high-density lipoprotein (HDL) particles, inflammation, and oxidative stress condition. To verify the effects of LA procedure, the current study is aimed at analyzing the effect of a single apheresis procedure with direct hemadsorption (DALI) and cascade filtration (MONET) on oxidative stress markers and HDL-related parameters. The study included eleven patients with familial hypercholesterolemia and hyperlipoproteinemia(a) treated with regular LA (DALI or MONET). We investigated the pre- and postapheresis concentration of the lipid-related oxidative stress markers 8-isoPGF2, oxLDL, TBARS, and PON-1. We also tracked potential changes in the main HDL apolipoproteins (ApoA-I, ApoA-II) and cholesterol contained in HDL subfractions. A single session of LA with DALI or MONET techniques resulted in a similar reduction of lipid-related oxidative stress markers. Concentrations of 8-isoPGF2 and TBARS were reduced by ~60% and ~30%, respectively. LA resulted in a 67% decrease in oxLDL levels along with a ~19% reduction in the oxLDL/ApoB ratio. Concentrations of HDL cholesterol, ApoA-I, ApoA-II, and PON-1 activity were also reduced by LA sessions, with more noticeable effects seen in the MONET technique. The quantitative proportions between HDL_2_ and HDL_3_ cholesterol did not change significantly by both methods. In conclusion, LA treatment with MONET or DALI system has a small nonselective effect on lowering HDL particles without any changes in the protein composition of these particles. Significant reduction in the level of oxidative stress parameters and less oxidation of LDL particles may provide an additional benefit of LA therapy.

## 1. Introduction

Atherosclerosis and related cardiovascular disease represent a major health problem in Western countries and constitute a leading cause of morbidity and mortality [1]. The relationship among elevated low-density lipoprotein (LDL) particles, their oxidation, and the progression of atherosclerosis is well recognized [2]. More recently, an increased lipoprotein(a) (lp(a)) level was identified as a major cardiovascular lipid-related risk factor [3, 4].

Lipid-lowering medications, diet, and lifestyle modification do not always achieve the intended and restrictive therapeutic goals and proper reduction of cardiovascular event rate [5]. Individuals with severe familial hypercholesterolemia (FH) and those with high levels of lp(a) may require extracorporeal treatment with lipoprotein apheresis (LA). Specific columns not only remove LDL particles and lp(a) but also affect the concentration of chylomicrons, very-low-density lipoproteins (VLDL), and high-density lipoproteins (HDL) [6]. Several techniques of LA are available. The elimination mechanism can either be based on precipitation (heparin-mediated extracorporeal LDL precipitation—H.E.L.P.), membrane filtration (Membrane Filtration Optimized Novel Extracorporeal Treatment—MONET), adsorption from whole blood (direct adsorption by polyacrylate/polyacrylamide—DALI), or immunoabsorption (IA).

Selective LA therapies, regardless of the technique used, have been shown to be safe and reduce the rate of adverse cardiac or vascular events [7–9]. Recent studies have suggested that the clinical benefits of LA may be associated with the impact of LA on inflammation and oxidative stress condition [10–12]. Published data have proved that increased oxidative stress in patients with FH enhanced the proatherogenic properties of LDL particles and participated in the acceleration of atherosclerosis [13]. However, investigations on the effect of single apheresis procedures on oxidative stress parameters are rare and remain inconclusive. While some have shown that LA treatment can decrease oxidative stress biomarkers [11, 14], others have demonstrated the enhancement of oxidative processes [15, 16].

Another interesting phenomenon is the effect of LA on HDL particles. The low HDL cholesterol phenotype observed in FH patients may also contribute to premature atherosclerosis. HDL particles possess multiple antiatherogenic functions related to their participation in reverse cholesterol transport, as well as antioxidant and anti-inflammatory capacity [17]. In this context, the depletion of HDL particles may be understood as a counterproductive result of LA. However, HDL particles are a very heterogeneous group of particles that differ in composition and functionality. Therefore, the assessment of the impact of LA only on the amount of HDL cholesterol does not reflect the potential changes in the composition and quality of these particles.

In the current study, we aimed to more fully explain the effect of apheresis by analyzing the effect of a single apheresis procedure using the MONET and DALI techniques on lp(a), lipid-related oxidative stress markers, including isoprostanes (8-iso-prostaglandin F2a), thiobarbituric acid reactive substances (TBARS), and oxidized LDL particles (oxLDL). In addition, we evaluated the level of major apolipoproteins contained in HDL particles (ApoA-I, ApoA-II), assessed the activity of the HDL-related antioxidant enzyme paraoxonase-1 (PON-1), and tracked potential changes in cholesterol in HDL_2_ and HDL_3_ subfractions.

## 2. Methods

### 2.1. Subjects

The study cohort consisted of eleven patients on regular LA therapy. The indication for LA in eight patients was FH with the inability to achieve LDL-C treatment goals using maximally tolerated lipid-lowering therapy. Three patients had isolated hyperlipoproteinemia(a) with an lp(a) level above 100 mg/dl and LDL-C levels on target. Regular LA treatment was performed at biweekly intervals using two methods, according to the patients' characteristics and indications, as previously described [18, 19]. Seven individuals were treated with the cascade filtration method (MONET) and four with the direct hemadsorption (DALI) technique. The anticoagulation was based on heparin and citrate infusion. Each procedure was designed and conducted to achieve LDL-C and lp(a) reduction of at least 60% and processed at least 45 ml of plasma volume per kg of body weight in MONET procedures or 1.5 l of blood volume in DALI sessions.

FH was diagnosed based on the Dutch Lipid Clinic Network Score, validated in the Polish population [20]. Before the initiation of LA, all subjects were treated with maximally tolerated lipid-lowering therapy to achieve recommended LDL-C treatment goals [5, 21]. The study was performed in accordance with the ethical guidelines of the 1975 Declaration of Helsinki and was approved by the Medical Ethics Committee of the Medical University of Gdańsk (Project code: 428/2018-2019). All of the participants provided written informed consent.

### 2.2. Laboratory Measurements

Blood samples were obtained from peripheral blood, in a fasting state directly before apheresis sessions and immediately after the apheresis procedure, as previously described [18]. The serum was separated after centrifugation at 1000 g for 15 min and was stored at −80°C until analysis.

The total cholesterol (TC) and triacylglycerols (TAG) were measured using commercially available enzymatic kits obtained from Pointe Scientific (Warsaw, Poland). The LDL-cholesterol (LDL-C) levels were measured using commercially available Direct LDL Kits from Abbott Laboratories (Chicago, United States). The HDL was isolated by the precipitation of apolipoprotein B-containing lipoproteins with heparin and manganese chloride, and the HDL-cholesterol (HDL-C) was measured in the supernatant using a kit obtained from Pointe Scientific. HDL_2_ and HDL_3_ subfractions were isolated by density gradient (HDL_2_:1.06-1.125, HDL_3_:1.125-1.25) ultracentrifugation in a Beckman Coulter TLA 120 2 [22]. lp(a) concentrations were measured using a commercially available lp(a) kit from Abbott Laboratories (Chicago, United States). The ApoB, ApoA-I, and ApoA-II concentrations were determined using the nephelometric method with antibodies obtained from Siemens Healthcare Diagnostics (Eschborn, Germany) with a Behring laser nephelometer. The paraoxonase-1 activity was measured in serum with paraoxon ethyl as the substrate, according to the procedure described earlier [23]. The concentration of TBARS was analyzed by fluorescence spectroscopy using a modified thiobarbituric acid-reactive substance [24]. oxLDL was analyzed using an enzyme immunoassay kit (EIAab, China), and 8-Iso-PGF2*α* was analyzed in plasma using an enzyme immunoassay kit (Cayman Chemical, USA).

### 2.3. Statistical Analysis

Statistical analyses were performed using STATISTICA software, version 13 (StatSoft, Kraków, Poland). The Shapiro–Wilk test was used to test the determined normality of the distribution of variables. The variables were expressed as mean ± SD (standard deviation) or as medians with 25th and 75th percentiles. The one-way analysis of variance (ANOVA) for related variables or the *Friedman test* was used to assess the changes in individual parameters due to apheresis sessions, and the multivariate ANOVA was used to assess the impact of the apheresis technique on these changes. Pearson's chi-squared test was used to compare categorical variables. *P* values below 0.05 were considered to be statistically significant.

## 3. Results

The demographic and clinical data are presented in Table 1. The detailed characteristics of all investigated patients (*n* = 11) are shown in Supplemental Table S1. Of the eleven patients undergoing lipoprotein apheresis treatment, 7 were diagnosed with hypertension, 2 were diagnosed with diabetes, and 5 were past smokers. Ten patients had coronary artery disease (CAD). Of the eleven, ten patients were administered a potent statin in combination with ezetimibe and one patient was stain naïve due to mitochondrial myopathy.

The two investigated apheresis techniques, MONET and DALI, were similarly effective in lowering LDL-C (by 62% and 67%, respectively) and lp(a) (by 60% and 74%, respectively) and resulted in a comparable reduction of TC (by 45% and 43%, respectively), non-HDL-C concentration (by 51% and 53%, respectively), and TG concentration (by 53% and 52%, respectively) (Table 2).

**Figure 1:**
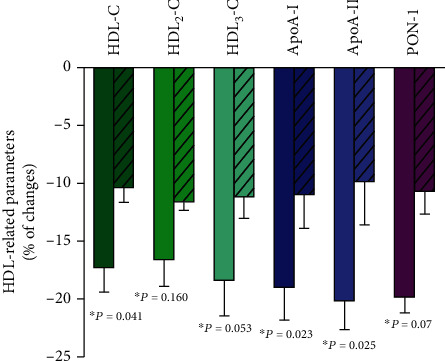
Percentage changes in HDL-related parameters due to the apheresis sessions. Bar graphs showing percentage changes in HDL-related parameters due to the MONET (open bar) and DALI (dashed bar) sessions. Values are presented as mean ± standard deviation. ^∗^The multivariate ANOVA for related variables was used to assess the impact of the method on the changes in individual parameters.

The HDL-C, ApoA-I, and ApoA-II concentrations and PON-1 activity were reduced with the MONET technique on an average by 17%, 19%, 20%, and 20%, respectively. The decrease in the above parameters with the DALI technique was approximately halved (Figure 1). The reduction of cholesterol concentration was similar for both HDL_2_ and HDL_3_ (~17% using the MONET technique and ~11% using the DALI technique). The quantitative proportions of HDL_2_ and HDL_3_ cholesterol did not change significantly due to the LA sessions, irrespective of the method used (Table 2).

**Figure 2:**
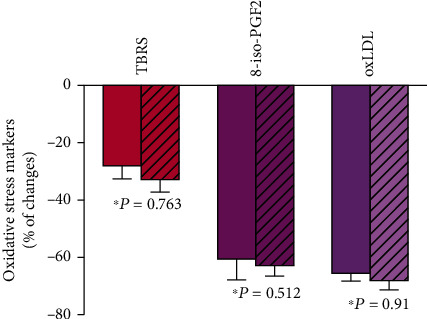
Percentage changes due to the apheresis sessions. Bar graphs showing percentage changes in oxidative stress markers due to the MONET (open bar) and DALI (dashed bar) session. Values are presented as mean ± standard deviation. ^∗^The multivariate ANOVA for related variables was used to assess the impact of the method on the changes in individual parameters.

A single session of LA using both the investigated techniques resulted in a significant reduction of oxidative stress marker levels, in addition to lowered lipid parameters. The 8-isoPGF2 concentration was reduced by 60% and 62%, TBARS concentration by 28% and 32%, and oxLDL concentration by 66% and 68% for the MONET and DALI techniques, respectively (Figure 2). In parallel, we noticed 21% and 17% reductions, respectively, in the oxLDL/ApoB ratio (Table 3).

## 4. Discussion

We have shown that the MONET and DALI techniques are similar in terms of the reduction of stress marker levels and proatherogenic lipoproteins. The obtained results are in line with previously published data [16] and confirmed the selectivity of atherogenic lipid removal, showing only a slight reduction in HDL cholesterol levels, which was more noticeable with the MONET technique. A decrease in HDL cholesterol of 12-20%, depending on the type of LA, is well-known and can be considered an unwanted effect of the treatment [25]. The participation of HDL particles in reverse cholesterol transport as well as their antioxidant and anti-inflammatory properties is thought to be protective in atherosclerosis. Nevertheless, HDL particles are a heterogeneous fraction. Accumulating evidence indicates that in the presence of systemic inflammation, HDL particles become dysfunctional, mainly as a result of the oxidation of their lipid and protein components, becoming cytotoxic and contributing to accelerated atherosclerosis [26]. Thus, the depletion of this subtype of particles would be beneficial even for patients at high cardiovascular risk. However, data on the impact of apheresis on individual HDL subfractions and their properties are scarce. Opole et al. analyzed 10 subjects treated with the H.E.L.P. system and 3 patients treated with the lipoprotein adsorption technique and showed selective removal of proinflammatory HDL particles. This was explained by a change in lipoprotein composition and surface charge resulting in a less negative cation, which may have enhanced its removal by LA [27]. Using the DALI technique, Orsoni et al. demonstrated that the highest reduction in HDL particles by LA was the result of a reduction in the HDL_2_ subfraction, which contained ~70% of total HDL ApoE [28]. Nevertheless, other studies conducted by these researchers showed no effect of apheresis on the ability of HDL particles to promote reverse cholesterol transport [29]. In our study, the decrease in serum ApoA-I, ApoA-II concentration, and activity of the HDL-linked antioxidant enzyme PON-1 was parallel to the decrease in HDL-C concentration. Moreover, we did not observe a change in the quantitative proportions of HDL_2_ and HDL_3_ cholesterol due to the apheresis sessions. Thus, it can be assumed that LA caused an unselective depletion of HDL subfractions and that this was more pronounced in the MONET technique. The smaller degree of HDL cholesterol reduction when using the DALI technique indicates that the adsorption techniques have a lower influence on HDL-related parameters.

Since oxidative stress is believed to play a major pathogenic role in vascular disease, the impact of apheresis on oxidative stress parameters appears to be clinically important, especially considering patients with FH and hyperlipoproteinemia(a) are characterized by the intensification of prooxidative processes [30]. Our study showed a clear decrease in oxidative stress parameters after a single apheresis session, which is in line with previous studies showing a significant reduction in free oxygen radicals and an increase in free oxygen radical defense [11, 14]. Nevertheless, not all studies have shown a positive impact of apheresis on systemic oxidative burden. Kopprasch et al., analyzing oxidant generation by phagocytes in whole blood and isolated leukocytes, demonstrated a transiently increased production of reactive oxygen species (ROS) following H.E.L.P. apheresis, while also suggesting a biochemical benefit of a single DALI treatment, namely, lower systemic oxidative burden in comparison to HELP and IA procedures [13].

In our work, we did not observe differences between the impact of the MONET and DALI techniques on lipid-related oxidative stress parameters, including a ~60% reduction in 8-isoPGF2 levels, which are the most valid in vivo lipid peroxidation biomarkers and exert proatherogenic function via their vasoconstrictive platelet-activating and mitogenic properties [31, 32]. We also noticed a decrease in oxLDL concentration. An increase in the oxidation of LDL due to prolonged intravascular residence time has been established as one of the key pathogenic mechanisms for the development of premature coronary lesions in hypercholesterolemia. In our work, along with the decrease in the oxLDL level, we noted reductions in the oxLDL/ApoB ratio, which may reflect the ratio of oxidatively modified LDL to total LDL particles. A decrease in LDL susceptibility to oxidation after lipid apheresis has been noted earlier [33]. Some researchers have speculated that exogenous removal of LDL induces changes in the chemical composition, such as an increase in the content of vitamin E, oleic acid, and arachidonic acid in LDL particles, which may lead to increased resistance against oxidation [34].

A computer simulation study by Donner et al., investigating the effect of dextran sulfate apheresis and HELP techniques on LDL oxidizability and performing, reported a decrease in the susceptibility of LDL to oxidation after apheresis session as the result of an altered ratio between freshly produced (less susceptible to oxidation) and older (more susceptible to oxidation) LDL particles. This speculation seems to be independent of the apheresis method and can also explain the lower ratio of oxidatively modified LDL to total LDL particles after MONET and DALI techniques observed in our study [35].

Our study has some limitations. The most important is the relatively small sample size. This is primarily because although LA treatment is fully reimbursed in Poland, only 4 centers offer such last-line therapy. To our knowledge, only 20 patients are currently undergoing regular LA in Poland. In conclusion, LA sessions, in addition to lowering the concentration of proatherogenic lipoproteins, have an acute, minor, nonselective effect on lowering HDL particles but do not change the protein composition of these particles and do not appear to affect their antioxidant properties associated with PON-1 activity. We observed a significant reduction in the level of oxidative stress parameters and demonstrated a reduction in the oxidation of LDL particles, which may provide additional benefit to LA therapy.

## Figures and Tables

**Table 1:** Demographic and clinical data.

Parameters	MONET (*n* = 7)	DALI (*n* = 4)	*P*
Gender (M/F)	4/3	2/2	0.472
Age (years)	59 ± 13	54 ± 6	0.953
Height (cm)	170 ± 10	169 ± 9	0.979
Weight (kg)	86 ± 24	86 ± 10	0.788
BMI (kg/m^2^)	29 ± 6	30 ± 6	0.486
HR (bpm)	61 ± 9	64 ± 5	0.271
SBP (mmHg)	124 ± 6	118 ± 11	0.978
DBP (mmHg)	72 ± 7	72 ± 4	0.472

Continuous values are presented as means ± standard deviation. Potential differences between MONET and DALI techniques were analyzed using ANOVA for unrelated variables or Pearson's chi-squared test for nominal data. BMI: body mass index; DBP: baseline diastolic blood pressure; HR: baseline resting heart rate; SBP: baseline systolic blood pressure.

**Table 2:** Impact of lipoprotein apheresis on plasma lipids and apolipoproteins.

Parameters	MONET			DALI		
	Preapheresis	Postapheresis	*P*	Preapheresis	Postapheresis	*P*
TC (mg/dl)	178 ± 63	92 ± 28	0.002^∗^	169 ± 63	94 ± 29	0.019^∗^
LDL-C (mg/dl)	136 ± 62	50 ± 26	<0.001^∗^	137 ± 68	50 ± 34	0.012^∗^
ApoB (g/l)	1.19 ± 0.35	0.53 ± 0.19	0.002^∗^	1.28 ± 1.62	0.53 ± 0.36	0.002^∗^
Non-HDL-C (mg/dl)	142 ± 61	61 ± 33	0.002^∗^	134 ± 73	63 ± 34	0.043^∗^
TAG (mg/dl)	188 (98-255)	78 (55-111)	0.017^∗∗^	182 (158-340)	85 (61-182)	0.067^∗∗^
lp(a) (mg/dl)	79 (12-116)	17 (5-60)	0.017^∗∗^	119 (32-274)	30 (8-71)	0.068^∗∗^
HDL-C (mg/dl)	36 ± 6	30 ± 6	<0.001^∗^	34 ± 8	31 ± 12	0.012^∗^
HDL_2_-C (mg/dl)	22 ± 3	19 ± 3	0.018^∗^	22 ± 7	20 ± 6	0.003^∗^
HDL_3_-C (mg/dl)	14 ± 3	11 ± 3	<0.001^∗^	12 ± 5	11 ± 5	0.015^∗^
HDL_2_-C/HDL_3_-C ratio	1.65 ± 0.15	1.7 ± 0.22	0.404^∗^	1.9 ± 0.45	1.9 ± 0.5	0.996^∗^
ApoA-I (g/l)	1.41 ± 0.28	1.15 ± 0.13	0.003^∗^	1.45 ± 0.27	1.26 ± 0.02	0.046^∗^
ApoA-II (g/l)	0.32 ± 0.06	0.25 ± 0.06	0.003	0.31 ± 0.08	0.30 ± 0.08	0.009
ApoB/ApoA-I	0.85 ± 0.29	0.48 ± 0.20	<0.001	1.00 ± 0.63	0.47 ± 0.36	0.02^∗^

Continuous values are presented as means ± standard deviation or as medians (25th-75th percentile). Potential differences between pre- and postapheresis results were analyzed using ^∗^ANOVA for related variables or ^∗∗^a nonparametric Friedman test.

**Table 3:** Impact of lipoprotein apheresis on plasma oxidative stress marker level.

Parameters	MONET			DALI		
	Preapheresis	Postapheresis	*P*	Preapheresis	Postapheresis	*P*
8-Isoprostane (pg/ml)	29 (19-93)	11 (8-18)	0.01^∗∗^	33 (27-40)	12 (9-26)	0.001^∗∗^
TBARS (*μ*mol/l)	3.1 ± 0.4	2.1 ± 0.1	0.001	2.8 ± 0.8	1.8 ± 0.4	0.027^∗^
oxLDL (ng/ml)	62 ± 21	20 ± 5	<0.001	63 ± 14	20 ± 5	0.005^∗^
oxLDL/ApoB (ng/mg)	57 ± 11	43 ± 16	0.008	60 ± 34	52 ± 34	0.008^∗^
PON-1 (U/l)	186 ± 94	146 ± 71	0.002	225 ± 65	191 ± 52	0.057^∗^

The values are presented as means ± standard deviation or as medians (25th and 75th percentile). Potential differences between pre- and postapheresis results were analyzed using ^∗^ANOVA for related variables or ^∗∗^a nonparametric Friedman test.

## Data Availability

The data (database in Excel) used to support the findings of this study are available from the corresponding author upon request (agnieszka.kuchta@gumed.edu.pl).

## Supplementary Materials

Supplementary Materials Table S1: detailed characteristics of investigated patients.
