# Supplementary material for: The Impact of Lipoprotein Apheresis on Oxidative Stress Biomarkers and High-Density Lipoprotein Subfractions
Source: Oxid Med Cell Longev. 2020 Aug 5;2020:9709542. doi: 10.1155/2020/9709542 (PMC7428943; doi:10.1155/2020/9709542)
Supplement: Supplementary Materials — Table S1: detailed characteristics of investigated patients. [file 9709542.f1.docx]

Table S1. Detailed characteristics of investigated patients

| **Patient's ID** | **1** | **2** | **3** | **4** | **5** | **6** | **7** | **8** | **9** | **10** | **11** |
| --- | --- | --- | --- | --- | --- | --- | --- | --- | --- | --- | --- |
| **Gender** | female | male | female | male | male | female | male | female | female | male | male |
| **Age [years]** | 61 | 66 | 58 | 49 | 49 | 79 | 65 | 54 | 60 | 56 | 35 |
| **Time on apheresis [months]** | 82 | 26 | 59 | 31 | 42 | 31 | 32 | 42 | 59 | 2 | 16 |
| **Smoking** | 0 | 1 | 1 | 1 | 0 | 1 | 0 | 0 | 0 | 0 | 0 |
| **Hypertension** | 1 | 1 | 1 | 0 | 0 | 1 | 1 | 0 | 1 | 0 | 1 |
| **Diabetes** | 0 | 0 | 0 | 0 | 0 | 1 | 0 | 0 | 1 | 0 | 0 |
| **CAD** | 1 | 1 | 1 | 1 | 1 | 1 | 1 | 0 | 1 | 1 | 1 |
| **Height [cm]** | 170 | 190 | 158 | 179 | 175 | 155 | 169 | 163 | 167 | 173 | 171 |
| **Weight [kg]** | 71 | 117 | 93 | 71 | 91 | 68 | 82 | 53 | 91 | 98 | 125 |
| **BMI [kg/m^2^]** | 25 | 32 | 37 | 22 | 30 | 28 | 29 | 20 | 33 | 33 | 43 |
| **HR baseline [bpm]** | 78 | 60 | 61 | 60 | 70 | 60 | 64 | 61 | 68 | 50 | 68 |
| **SBP baseline [mmHg]** | 130 | 119 | 110 | 110 | 119 | 135 | 127 | 119 | 135 | 122 | 114 |
| **DBP baseline [mmHg]** | 58 | 72 | 71 | 70 | 70 | 80 | 70 | 77 | 78 | 74 | 78 |
| **DALI/**  **MONET** | MONET | MONET | DALI | DALI | DALI | MONET | MONET | MONET | DALI | MONET | MONET |
| **Lipid-lowering medications** | Rosuvastatin 40 mg + Ezetimibe 10 mg | Atorvastatin 80 mg + Ezetimibe 10 mg | Atorvastatin 80 mg + Ezetimibe 10 mg | Rosuvastatin 40 mg + Ezetimibe 10 mg | Rosuvastatin 40 mg + Ezetimibe 10 mg | Rosuvastatin 20 mg + Ezetimibe 10 mg | Rosuvastatin 40 mg + Ezetimibe 10 mg | none (mitochondrial miopathy) | Atorvastatin 40 mg + Ezetimibe 10 mg | Rosuvastatin 40 mg + Ezetimibe 10 mg | Rosuvastatin 40 mg + Ezetimibe 10 mg |

*Abbreviations: BMI – body mass index, CAD – coronary artery disease, DALI - direct adsorption by polyacrylate/polyacrylamide, DBP – diastolic blood pressure, HR – heart rate, MONET - Membrane Filtration Optimized Novel Extracorporeal Treatment, SBP – systolic blood pressure,*
